# Supplementary material for: Pulsatilla koreana Nakai Extract Attenuates Atopic Dermatitis-like Symptoms by Regulating Skin Barrier Factors and Inhibiting the JAK/STAT Pathway
Source: Int J Mol Sci. 2025 Mar 25;26(7):2994. doi: 10.3390/ijms26072994 (PMC11988913; doi:10.3390/ijms26072994)
Supplement: Supplementary file 1 [file ijms-26-02994-s001.zip › ijms-3403903-supplementary.pdf]

Supplemetnary Figure legends

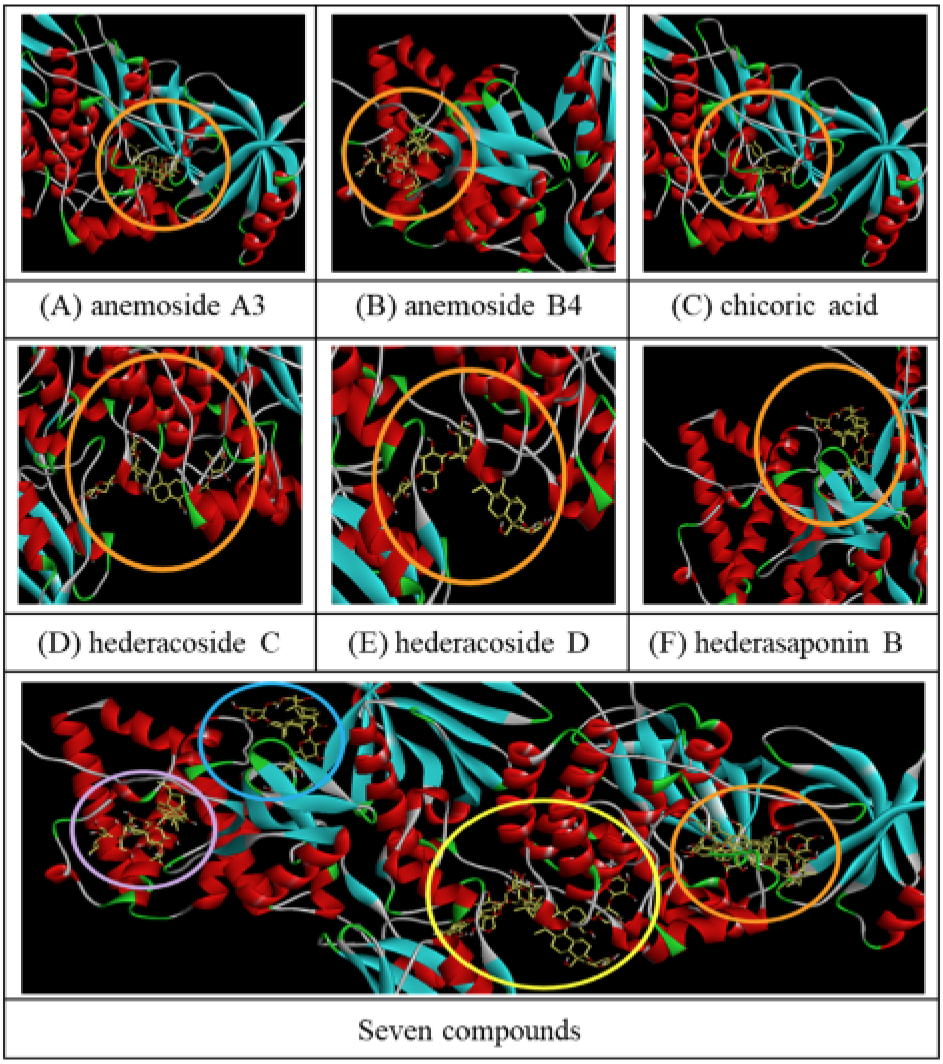

Scheme S1. Docking analysis results of JAK1 (A-F) The predicted results of docking analysis betwwen the main compounds of PKN and JAK1 were visualized. Orange circles indicated the binding site of each compound. (G) The binidng sites of seven major compounds were visualized throuhout the JAK1 protein. The orange circle is where AH, AA3 and CC bind, the protein kinase 2 domain that interferece with ATP activity of JAK kinases. The purple and blue circles are the binding sites for AB4 andHB, respectively, and the FERM-SH2 domain, which is the binding site for cytokines and iterferon receptors. AH, Alpha-hederin; AA3, Anemoside A3; AB4, Anemoside B4; CC, Chicoric acid; HC, Hedera-coside C; HD, Hederacoside D; HB, Hederasaponin B.

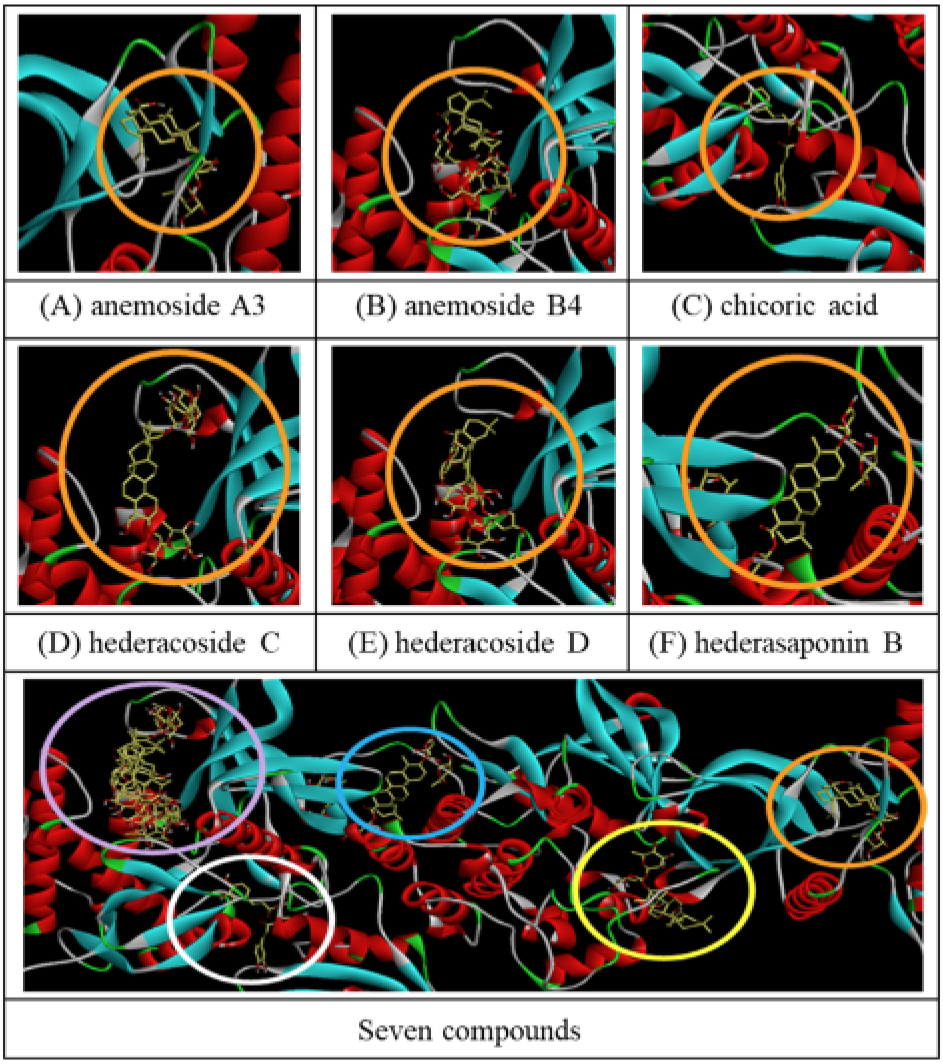

Scheme S2. Docking analysis results of JAK2 (A-F) The predicted results of docking analysis between the main compounds of PKN and JAK2 were visualized. Orange cir-cles indicate the binding site of each compound. (G) The binding sites of seven major com-pounds were visualized throughout the JAK2 protein. The orange circle is where AH binds, the protein kinase 2 domain that interferes with ATP activity. The yellow circle is where AA3 binds, the protein kinase 1 domain known to regulate the activity of JAK kinases. Purple circles (AB4, HC, HD), blue circles (HB), and white circles (CC) are all located in the FERM-SH2 domain. AH, Alpha-hederin; AA3, Anemoside A3; AB4, Anemoside B4; CC, Chicoric acid; HC, Hedera-coside C; HD, Hederacoside D; HB, Hederasaponin B.

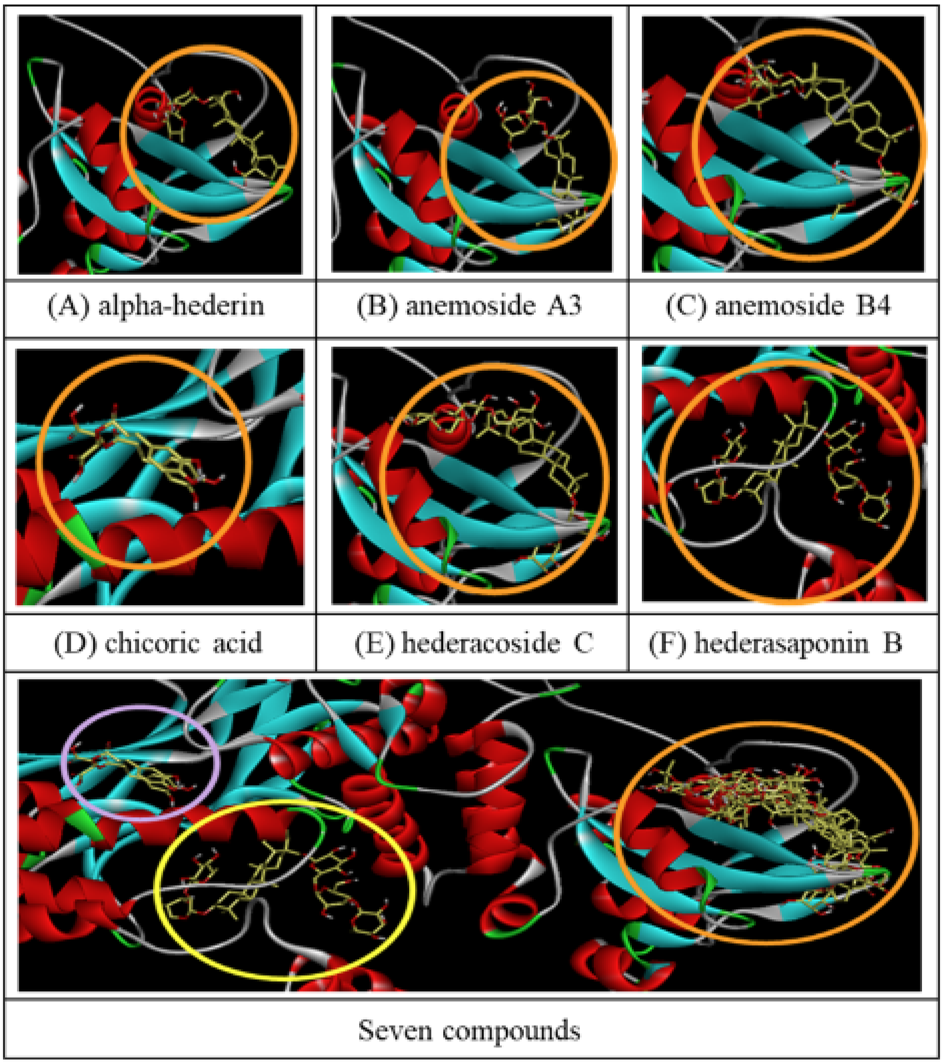

Scheme S3. Docking analysis results of STAT3 (A-F) The predicted results of docking analysis between the main compounds of PKN and STAT3 were visualized. Orange circles indicate the binding site of each compound. (G) The binding sites of seven major compounds were visualized throughout the STAT3 protein. The orange circle, where AH, AA3, AB4, HC, and HD bind, is the SH2 domain and is related to dimer formation of STAT3. The purple circle where CC binds is a coiled-coil domain that performs phosphorylation, and the yellow circle where HB binds is a DNA-binding domain that binds to DNA. AH, Alpha-hederin; AA3, Anemoside A3; AB4, Anemoside B4; CC, Chicoric acid; HC, Hederacoside C; HD, Hederaco-side D; HB, Hederasaponin B.

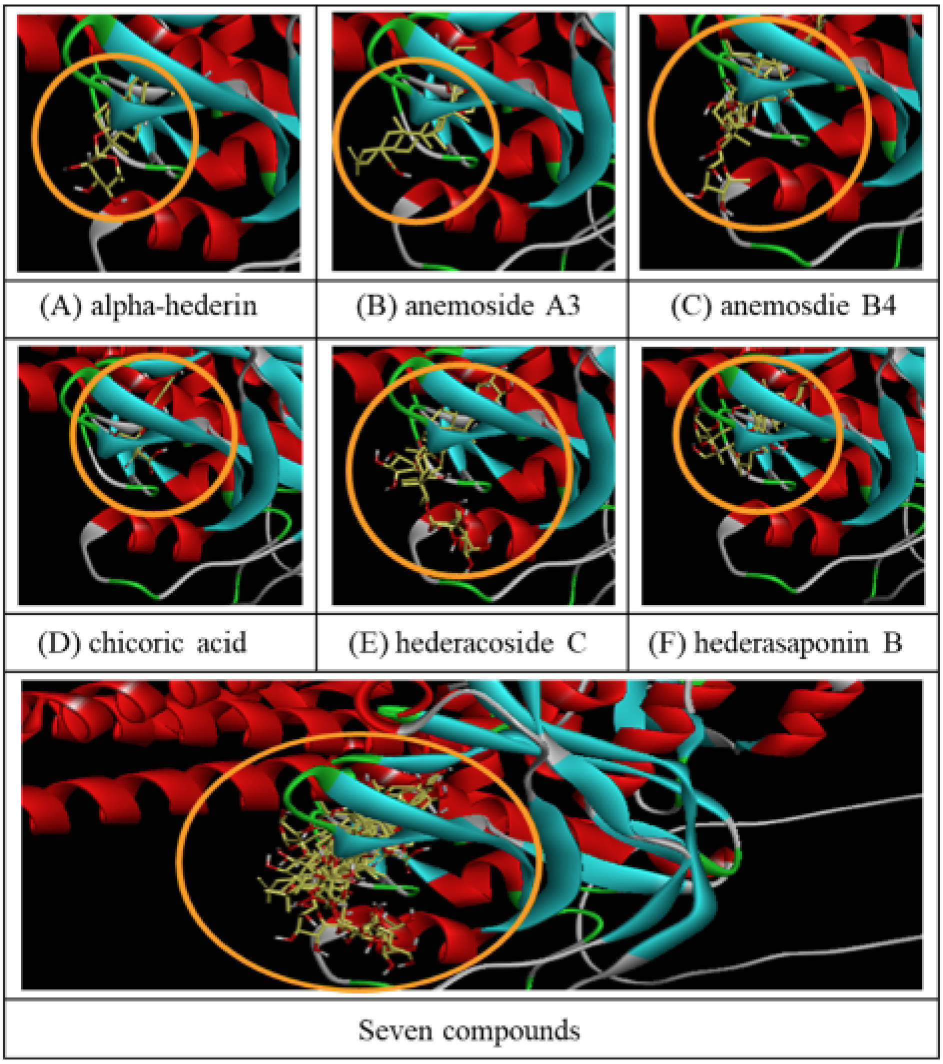

Scheme S4. Docking analysis results of STAT6 (A-F) The predicted results of docking analysis between the main compounds of PKN and STAT6 were visualized. Orange circles indicate the binding site of each compound. (G) The binding sites of seven major compounds were visualized throughout the STAT6 protein. All compounds bind to the DNA-binding domain, which inhibits the transcription factor role of STAT6.
